# Fiberoptic Bronchoscopy in Thyroid Carcinoma With Tracheal Invasion

**DOI:** 10.1155/DTE.4.113

**Published:** 1998

**Authors:** Haruya Koshiishi, Kunio Toriya, Osamu Ozaki, Kunihiko Ito, Chimori Konaka, Harubumi Kato

**Affiliations:** 1 Department of Surgery Tokyo Metropolitan Otsuka Hospital 2-8-1, Minamiotsuka Toshima-ku Tokyo 170 Japan; 2 Department of Surgery Ito Hospital Tokyo Japan; 3 First Department of Surgery Tokyo Medical College Tokyo Japan

## Abstract

Preoperative bronchoscopic findings of thyroid carcinoma with tracheal invasion were examined and compared with the histopathological findings. Tracheal sleeve resection was performed in 20 cases. The bronchoscopic findings were classified into 5 types: confirmed tumor in the tracheal lumen (5 cases), extramural compression of the trachea plus mucosal change (9 cases), extramural compression of the trachea (2 cases), mucosal changes only (3 cases), normal findings (1 case). Pathological findings revealed that the extent of invasion in the tracheal wall varied according to each of the above bronchoscopic types. The number of tracheal rings with adventitial invasion averaged 0.8 (maximum 2) more than preoperative bronchoscopic findings of the number of tracheal rings with mucosal invasion. This study demonstrated the necessity of resective 2 more tracheal rings than is indicated by the bronchoscopic findings.


*Diagnostic and Therapeutic Endoscopy*, Vol. 4, pp. 113–118
Reprints available directly from the publisher
Photocopying permitted by license only

© 1998 OPA (Overseas Publishers Association)
Amsterdam B.V. Published under license
under the Harwood Academic Publishers imprint,
part of The Gordon and Breach Publishing Group.
Printed in Singapore.

### Fiberoptic Bronchoscopy in Thyroid Carcinoma with Tracheal Invasion

HARUYA KOSHIISHI[a,*], KUNIO TORIYA[a], OSAMU OZAKI[b], KUNIHIKO ITO[b], CHIMORI KONAKA[c] and HARUBUMI KATO[c]

[a]*Department of Surgery, Tokyo Metropolitan Otsuka Hospital, 2-8-1, Minamiotsuka, Toshima-ku, Tokyo 170, Japan;* [b]*Department of Surgery, Ito Hospital,* [c]*First Department of Surgery, Tokyo Medical College, Tokyo, Japan*

*(Received 31 March 1997; Revised 15 July 1997; In final form 25 August 1997)*

**Preoperative bronchoscopic findings of thyroid carcinoma with tracheal invasion were examined and compared with the histopathological findings. Tracheal sleeve resection was performed in 20 cases. The bronchoscopic findings were classified into 5 types: confirmed tumor in the tracheal lumen (5 cases), extramural compression of the trachea plus mucosal change (9 cases), extramural compression of the trachea (2 cases), mucosal changes only (3 cases), normal findings (1 case). Pathological findings revealed that the extent of invasion in the tracheal wall varied according to each of the above bronchoscopic types. The number of tracheal rings with adventitial invasion averaged 0.8 (maximum 2) more than preoperative bronchoscopic findings of the number of tracheal rings with mucosal invasion. This study demonstrated the necessity of resective 2 more tracheal rings than is indicated by the bronchoscopic findings.**

*Keywords:* Thyroid carcinoma, Tracheal invasion, Bronchofiberscopy

### INTRODUCTION

One of the most important prognostic factor in thyroid carcinoma is tracheal invasion. Although recently tracheal sleeve resection is frequently performed in cases of differentiated thyroid carcinoma with tracheal invasion, it is difficult to accurately evaluate the extent of tumor invasion in the tracheal wall and the longitudinal extent of tumor preoperatively. In order to evaluate the reliability of bronchofiberscopy in thyroid carcinoma with tracheal invasion, we compared the bronchoscopic findings with the pathological findings of resected specimens in cases of tracheal sleeve resection.

### MATERIAL AND METHODS

The 20 cases of thyroid carcinoma with tracheal invasion resected at our institution from 1985 to 1994 in which preoperative bronchoscopy were entered into this study. Circumferential sleeve

* Corresponding author.

resection of the trachea was performed in all cases. The patients consisted of four men and 16 women with a mean age of 52.2 years (range: 29–75 years). These were 18 papillary carcinomas and two follicular carcinomas. A single operation was performed in 16 cases and reoperation for local recurrence was performed in four. Total thyroidectomy was performed in 17 cases, subtotal thyroidectomy in two, and lobectomy in one. Three to eight tracheal rings (average 4.85 rings) were resected in all cases. The resected tracheal specimens were examined histologically. One longitudinal and one circumferential specimen showing invasion was cut out of the trachea and hematoxilin–eosin-stained sections were prepared for microscopic examination. The grade of invasion within the tracheal wall and the longitudinal extent of invasion in the trachea was assessed microscopically in each specimen.

### RESULTS

The bronchoscopic findings of the 20 cases were classified into 5 types (Fig. 1). The main findings of the mucosal changes of types II and IV were severe redness and vascular engorgement by tumor invasion and in many cases it extended circumferentially not only near the tumor side but also to the contra-lateral side. In one case of type V, tracheal invasion was determined by intraoperative findings.

Pathological findings revealed that the grade of invasion within the tracheal wall varied according to the type of bronchoscopic findings (Table I). In all cases of types I–III, tracheal wall invasion extended to the submucosal or mucosal layer. In contrast, in three cases of types IV and V, tracheal wall invasion was limited to the cartilage layer, and in only one case was invaded to

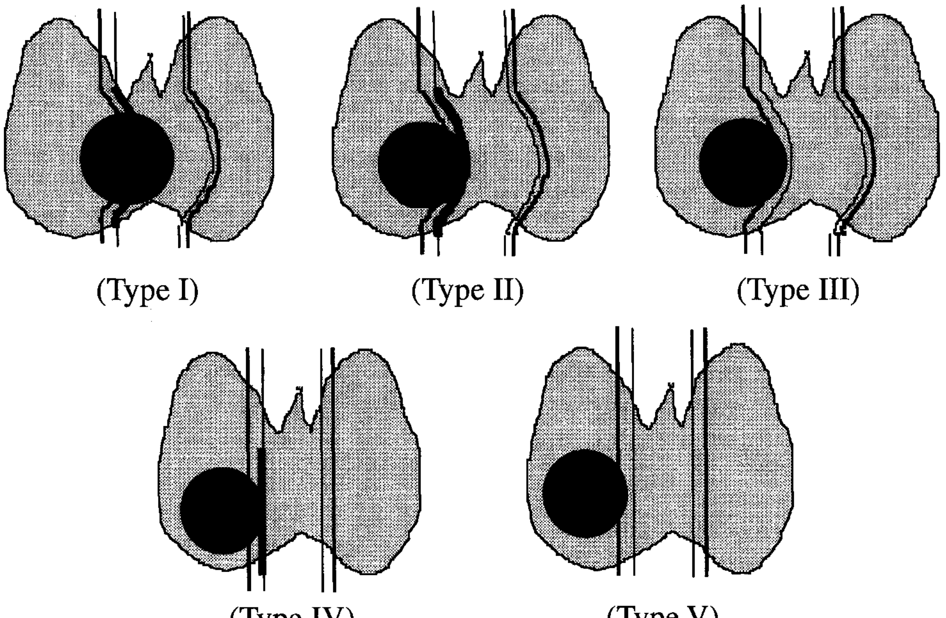

FIGURE 1 The classification of bronchoscopic findings in thyroid carcinoma with tracheal invasion. Type I: Confirmed tumor in the tracheal lumen. Type II: Extramural compression of the trachea plus mucosal change. Type III: Extramural compression of the trachea. Type IV: Mucosal change only. Type V: Normal findings.

TABLE I Correlation between BF type and tumor invasion of the tracheal wall

| BF type | Tumor invasion to the tracheal wall | | | | |
|---|---|---|---|---|---|
| | Mucosal layer | Submucosal layer | Cartilage layer | Extra-cartilage layer | Total |
| I | 5 | 0 | 0 | 0 | 5 |
| II | 3 | 6 | 0 | 0 | 9 |
| III | 0 | 2 | 0 | 0 | 2 |
| IV | 0 | 1 | 0 | 2 | 3 |
| V | 0 | 0 | 1 | 0 | 1 |
| Total | 8 | 9 | 1 | 2 | 20 |

($P < 0.01$).

TABLE II The average length of invaded tracheal rings between bronchoscopy and pathology

| BF type | Resected tracheal rings | Invaded rings based on bronchoscopical findings | Invaded rings based on pathological findings |
|---|---|---|---|
| I | 4.6 (4–5) | 3.7 (2–5) | 4.2 (4–5) |
| II | 5.5 (3–8) | 3.1 (2–4) | 3.9 (2–6) |
| III | 4 (4) | 3 (2–4) | 3 (3) |
| IV | 4 (3–5) | 2 (1–3) | 3 (2–4) |
| V | 4 (4) | 0 | 4 (4) |
| Total | 4.8 (3–8) | 2.9 (0–5) | 3.7 (2–6) |

(Minimum–Maximum).

the submucosal layer. When extramural compression of the trachea was recognized, pathological findings revealed a high grade of invasion within the tracheal wall.

The average number of invaded tracheal rings was compared in terms of bronchoscopic and pathological findings (Table II). Assessment of longitudinal extent of carcinoma showed invasion of on average of 2.9 rings (0–5 rings) by bronchoscopic findings, but on average of 3.7 rings (2–6 rings) by pathological findings in all cases, regardless of bronchoscopic type. The average number of resected tracheal rings was 4.8 (3–8 rings), and the cut end of the trachea was completely free of carcinoma tissue in all cases.

Bronchoscopic findings and pathological findings of the types I–IV are presented (Figs. 2–5).

### DISCUSSION

Recently neck soft X-ray, computed tomography (CT), magnetic resonance imaging (MRI) and fiberoptic bronchoscopy are generally performed preoperatively for thyroid carcinoma with tracheal invasion. Operative indications and methods are decided by the results of these examinations. In previous reports, the accurate diagnostic rate (sensitivity) for tracheal invasion was 25–85% in bronchofiberscopy, 60–100% in X-ray procedures including CT scans, especially invasion that is limited to the extra cartilaginous layer in tracheal wall is not identified successfully by any examinations [1–3].

Tracheal invasion was evaluated accurately in bronchoscopic types I and II, but in types III and V correct evaluation of tracheal invasion was difficult. Differential diagnosis between tracheal invasion and bronchitis is difficult in type IV. However, since hypervascularization and vascular engorgement of tracheal mucosa are characteristic of type IV, tracheal invasion could be diagnosed by careful observation of the vascular findings. Among all bronchoscopic types the sensitivity of tracheal invasion by the bronchoscopy alone was about 85%.

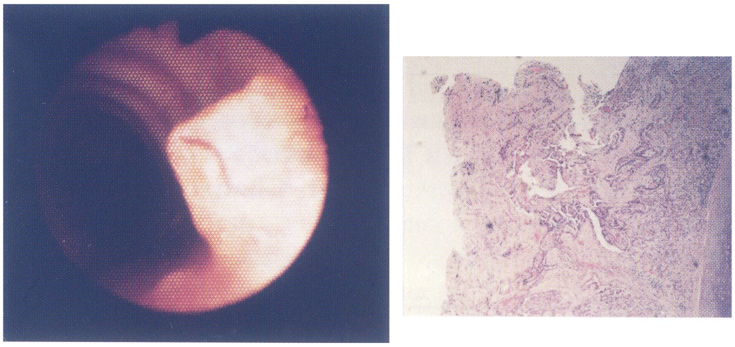

FIGURE 2 *BF type I: Confirmed tumor in the tracheal lumen.* Bronchoscopical findings reveal irregular tumor with vascular engorgement in the tracheal lumen (left). Pathological findings revealed papillary adenocarcinoma invaded to the mucosal layer (right).

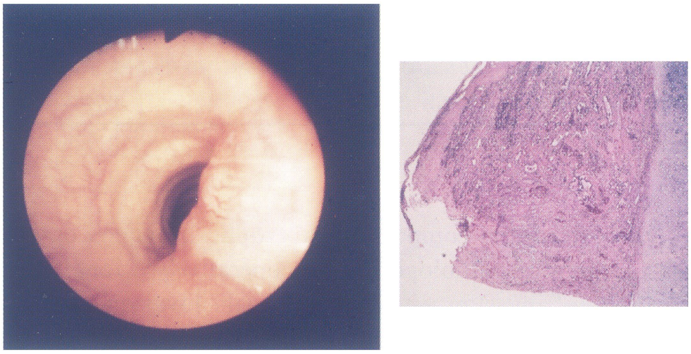

FIGURE 3 *BF type II: Extramural compression of the trachea plus mucosal change.* Bronchoscopical findings reveal extramural compression and vascular engorgement at the surface of tracheal mucosa (left). Pathological findings revealed papillary adenocarcinoma invaded to the mucosal layer (right).

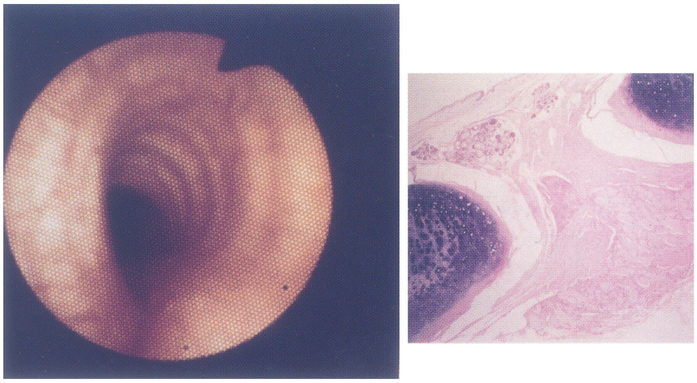

FIGURE 4 *BF type III: Extramural compression of the trachea.* Bronchoscopical findings reveal mild extra-luminal compression of trachea from right side (left). Pathological findings revealed follicular adenocarcinoma invaded to the cartilage layer (right).

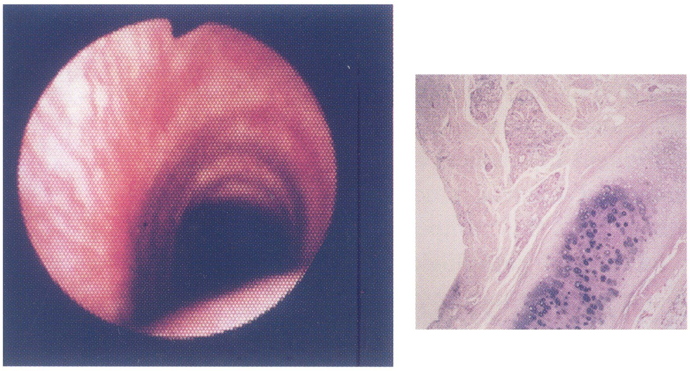

FIGURE 5 *BF type IV: Mucosal change only.* Bronchoscopical findings reveal severe vascular engorgement of the tracheal mucosa (left). Pathological findings revealed papillary adenocarcinoma invaded to the submucosal layer (right).

The bronchoscopic findings of primary lung cancer are classified into 5 types in The General Rules for Clinical and Pathological Recording of Lung Cancer [4]. Some patterns of the tumor growth form and bronchoscopic findings in this classification are the same as those of thyroid carcinoma with tracheal invasion. The mucosal changes recognizable bronchoscopically findings, especially vascular engorgement, redness and swelling are findings common to lung cancer and some cases of thyroid carcinoma with tracheal invasion in types II and IV [5].

Effectiveness of bronchofiberscopy for pre- and postoperative management of esophageal carcinoma has been reported. The bronchoscopic findings of esophageal carcinoma have been classified into 5 types, as follows: type I, tracheobronchial obstruction or stenosis due to the exposed tumor of esophageal carcinoma; type II, both protrusion of tracheobronchial wall due to the extrinsic esophageal tumor and abnormal findings of the mucosa; type IIIa, only protrusion of tracheobronchial wall due to the extrinsic esophageal tumor; type IIIb, only abnormal findings of mucosa; type IV, almost normal findings. These findings concerning tumor resectability in esophageal cancer with tracheal invasion have been reported [6,7]. In thyroid carcinoma with tracheal invasion, many cases classified bronchoscopically type I or II were found to be unresectable. Unresectable cases of esophageal carcinoma with tracheal invasion have same bronchoscopic findings as thyroid carcinoma. Cases of esophageal carcinoma in which bronchoscopie reveals mucosal changes without extramural compression (type IIIb) have little tracheal invasion [6,7], but in the present report, three cases of thyroid carcinoma (15%) in which same bronchoscopie have tracheal invasion. The reason of this difference is thought to be related to the tumor invasion site (thyroid: in the cartilage, esophagus: in the membranous portion) and tumor character (thyroid: slow growth, esophagus: rapid growth).

The indications and methods of tracheal resection in cases of thyroid carcinoma with tracheal invasion must be carefully decided with consideration of the tumor extent, the degree of differentiation and molecular biological characteristic [3,8–11]. We perform circumferential sleeve resection of the trachea, followed by end-to-end anastomosis whenever feasible excepting in cases of anaplastic carcinoma in our institution. Circumferential sleeve resection of trachea is essential because once the carcinoma tissue has invaded the submucosal area, circumferential tracheal invasion occurs [8]. In the present report, results of bronchoscopic findings that mucosal change extends circumferentially in the tracheal lumen in many cases of type II and IV recommends Ozaki's report.

Previous reports indicate that although it is important to resect the carcinoma tissue completely and safely, it can be difficult to accurately evaluate the extent of invasion within the tracheal wall based on preoperative examination and intraoperative findings [1,8,12]. Bronchofiberscopic examination is evaluated comparatively for accurate diagnosis of extent of tracheal invasion without a specific type of tracheal mucosal invasion. In the present report, the number of invaded tracheal rings recognized pathologically was an average of 0.8 more rings (maximum 2 rings) than estimated by preoperative bronchoscopy. The present study demonstrated the necessity of resecting 2 more tracheal cartilage rings than is indicated by the bronchoscopic findings.

### References

[1] Kikuchi, K., Kobayashi, T., Watanabe, M. *et al.* Tracheal resection in advanced thyroid carcinoma. *Operation* 1992; **46**: 91–96 (in Japanese).

[2] Hamada, H., Sasaki, F., Hata, Y. *et al.* A clinical study of differentiated thyroid carcinoma resected with the larynx and the trachea. *Jap. Clinic. Surg.* 1990; **51**: 23–28 (in Japanese).

[3] Kurihara, T., Ishida, T., Kurozumi, M. *et al.* Clinicopathological study of thyroid carcinoma with combined resection of the trachea. *Jpn. Cancer Ther.* 1991; **26**: 65–74 (in Japanese).

[4] The Japan Lung Cancer Society. General rule for clinical and pathological record of lung cancer (4th Edition). Kanehara publication, Tokyo, 1995 (in Japanese).

[5] Kato, H. and Horai, Y. A colour atras of endoscopic diagnosis in early stage lung cancer. Wolfe Publication, 1992: 35.

[6] Tanimura, H., Tomoyasu, H., Banba, J. *et al.* Fiberoptic bronchoscopy and computed tomography in esophageal carcinoma with tracheobronchial invasion. *J. Jap. Bronchology* 1989; **11**: 9–14 (in Japanese).

[7] Tanimura, H. Fiberoptic bronchoscopy and therapeutic method and prognosis in advanced esophageal carcinoma with tracheobronchial invasion. *J. Jap. Bronchology* 1990; **12**: 73–81 (in Japanese).

[8] Ozaki, O., Sugino, K., Mimura, T. *et al.* Surgery for patients with thyroid carcinoma invading the trachea; circumferential sleeve resection followed by end-to-end anastomosis. *Surgery* 1995; **117**: 268–271.

[9] Kure, Y., Sugino, K., Iwasaki, H. *et al.* The excisional range of tracheal infiltration of the thyroid cancer. *J. Jap. Surgical Soc.* 1991; **92**: 1694–1699 (in Japanese).

[10] Tsumori, N., Nakao, K., Miyata, M. *et al.* Clinicopathologic study of thyroid carcinoma infiltrating the trachea. *Cancer* 1985; **56**: 2843–2848.

[11] Nakaoka, Y., Takami, H., Moroda, N. *et al.* The biological malignancy of tracheally invading thyroid carcinoma. *J. Jap. Bronchology* 1989; **11**: 113–119 (in Japanese).

[12] Ishihara, T., Yamazaki, S., Kobayashi, K. *et al.* Resection of the trachea infiltrated by thyroid carcinoma. *Ann. Surg.* 1982; **195**: 496–500.

